# Supplementary material for: Fruit flies can learn non-elemental olfactory discriminations
Source: Proc Biol Sci. 2020 Nov 11;287(1938):20201234. doi: 10.1098/rspb.2020.1234 (PMC7735272; doi:10.1098/rspb.2020.1234)

Figure S1 : Performance Indices from individual groups of flies trained with a Paired protocol, Unpaired protocol and the Relative PI computed as the difference between two subsequently tested paired and unpaired groups. 1 cycle and 5 cycles PI were compared to assess the impact of repetition on flies calculated performances. Data are plotted as boxplots. the middle line represents the median, the upper and lower limits of the box are the 25 and 75 percent quantiles. The whiskers are the maximum and minimum values of the data that are respectively within 1.5 times the interquartile range over the 75th percentile and under the 25th percentile. Raw data are superimposed as jittered dots. “n.s” stands for “non-significant”, * indicates $p < 0.05$, ** indicates $p < 0.01$ after a t.test.

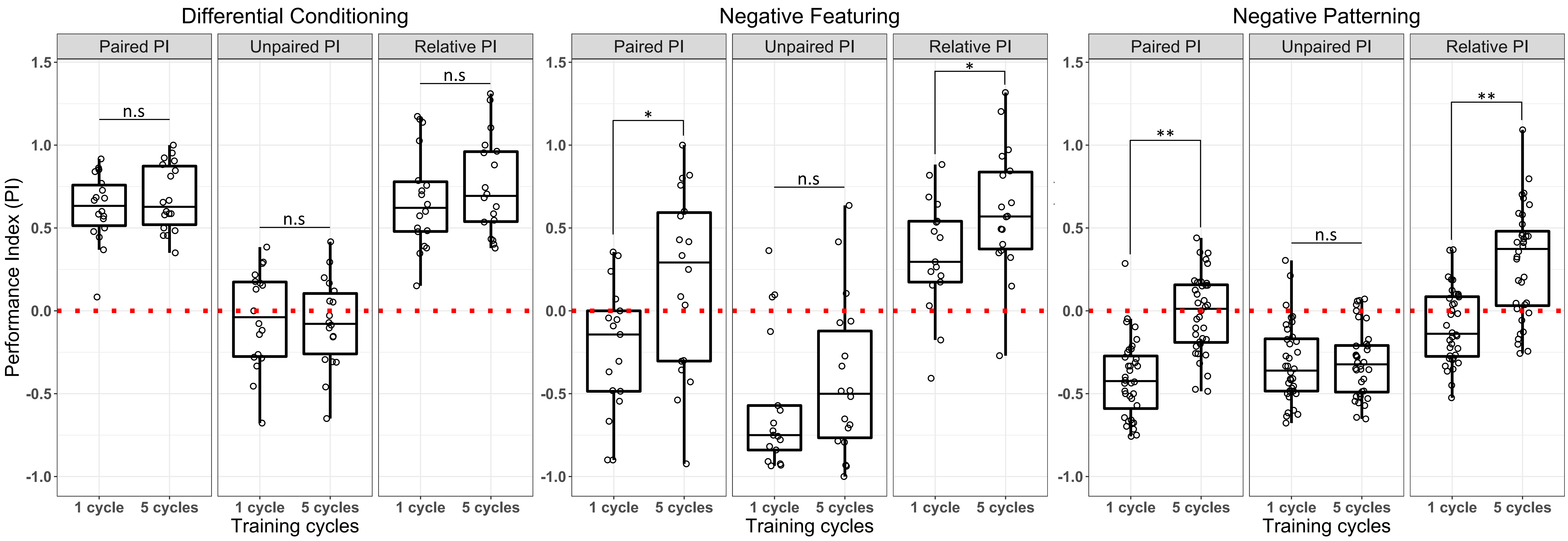

Figure S2 : Relative performance indices (PI) of flies trained in a Negative Patterning discrimination with alternative odourants (D = Isoamyl Acetate, E = Ethyl Butyrate), computed as the difference between paired and unpaired scores. Data are plotted as boxplots. The middle line represents the median while the upper and lower limits of the box are the 25 and 75 percent quantiles. The whiskers are the maximum and minimum values of the data that are respectively within 1.5 times the interquartile range over the 75th percentile and under the 25th percentile. Raw data are superimposed as jittered dots. ** indicates $p < 0.01$ after a 2-ways ANOVA

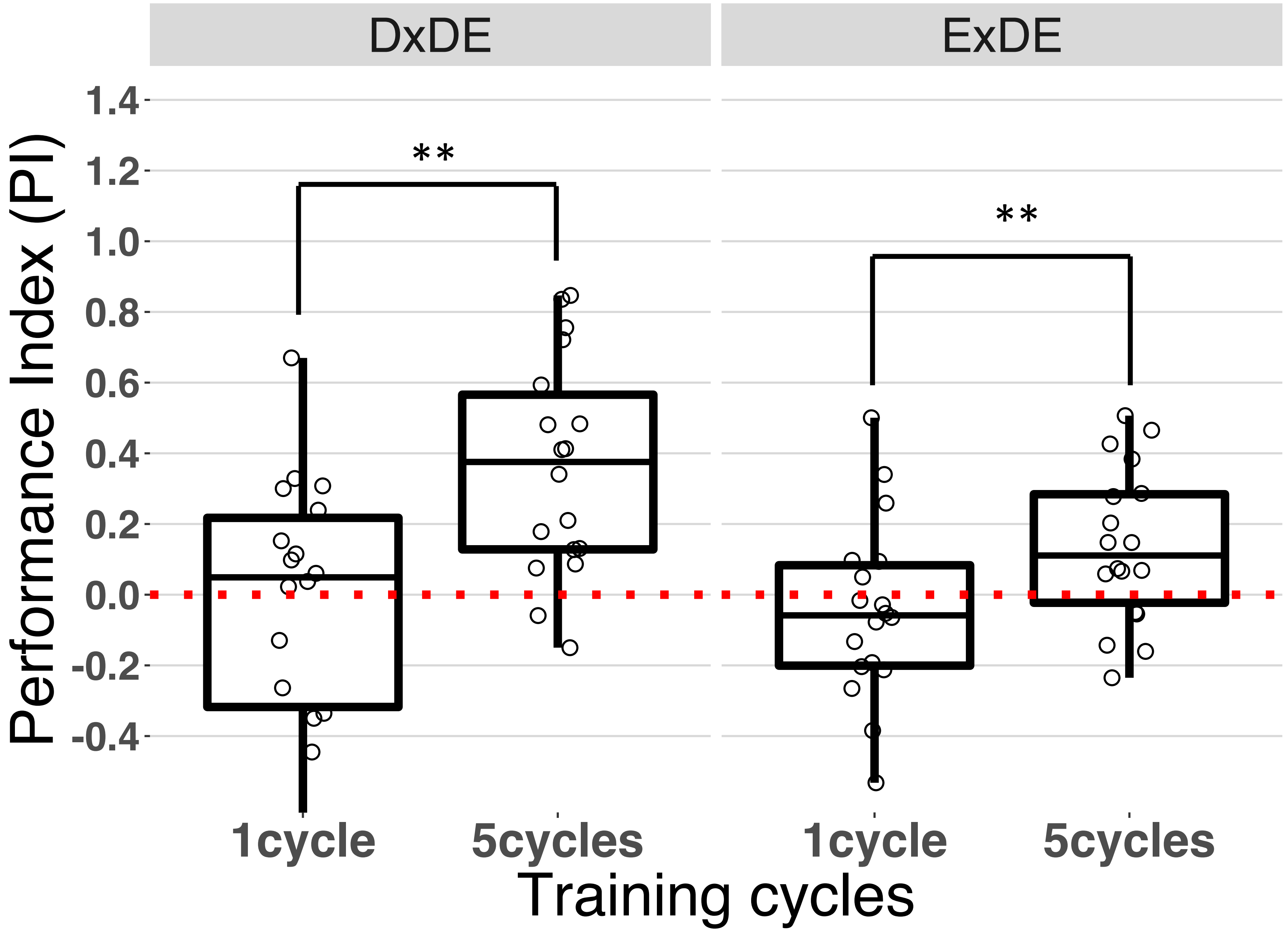

Figure S3: Relative Performance indices (PI) of flies trained either with a Differential Conditioning or Negative Feature discrimination, with 1 or 5 conditioning cycles, using B (4-Methylcyclohexanol) as CS+ and A (3-Octanol) as CS-. Data are plotted as boxplots. The middle line represents the median while the upper and lower limits of the box are the 25 and 75 percent quantiles. The whiskers are the maximum and minimum values of the data that are respectively within 1.5 times the interquartile range over the 75th percentile and under the 25th percentile. Raw data are superimposed as jittered dots.

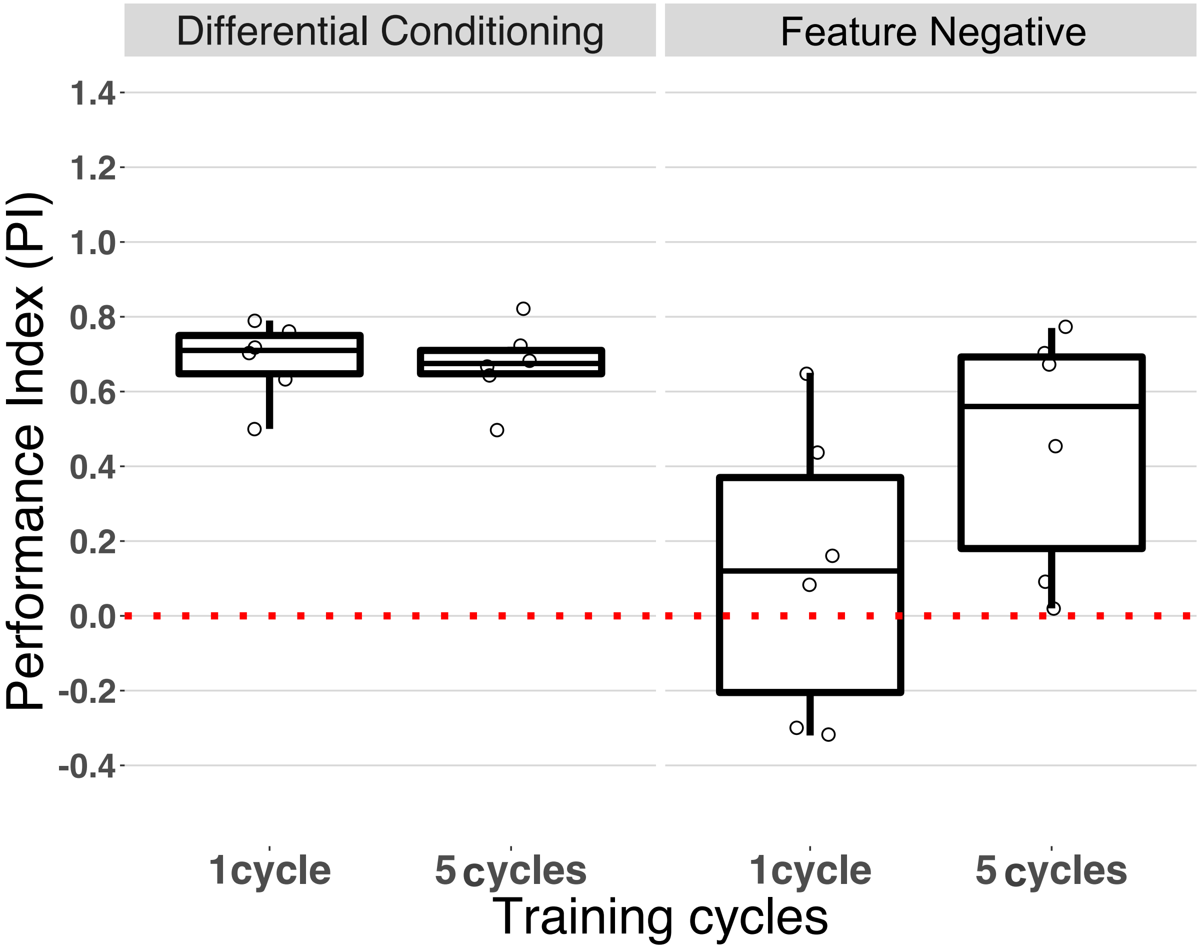

Supplement: Supplementary Figures S1 - S3 [file rspb20201234supp1.pdf]
